# Co-Administration of Proton Pump Inhibitors May Negatively Affect the Outcome in Inflammatory Bowel Disease Treated with Vedolizumab

**DOI:** 10.3390/biomedicines12010158

**Published:** 2024-01-11

**Authors:** Kata Szemes, Nelli Farkas, Zoltan Sipos, Renata Bor, Anna Fabian, Zoltan Szepes, Klaudia Farkas, Tamas Molnar, Eszter Schafer, Tamas Szamosi, Agnes Salamon, Aron Vincze, Patricia Sarlos

**Affiliations:** 1 Division of Gastroenterology, First Department of Medicine, Medical School, University of Pécs, 13 Ifjúság Street, 7624 Pecs, Hungary; 2 Institute of Bioanalysis, Medical School, University of Pécs, 7624 Pecs, Hungary; 3 First Department of Medicine, University of Szeged, 6720 Szeged, Hungary; 4 Department of Gastroenterology, Hungarian Defence Forces Military Hospital, 1134 Budapest, Hungary; 5 Balassa János Hospital, 7100 Szekszárd, Hungary; 6 Institute for Translational Medicine, Medical School, University of Pécs, 7624 Pecs, Hungary

**Keywords:** proton pump inhibitor, vedolizumab, inflammatory bowel disease

## Abstract

Concomitant medications may alter the effect of biological therapy in inflammatory bowel disease. The aim was to investigate the effect of proton pump inhibitors on remission rates in patients with inflammatory bowel disease treated with the gut-selective vedolizumab. Patients from the Hungarian nationwide, multicenter vedolizumab cohort were selected for post hoc analysis. Primary outcomes were the assessment of clinical response and endoscopic and clinical remission at weeks 14 and 54. Secondary outcomes were the evaluation of the combined effect of concomitant steroid therapy and other factors, such as smoking, on remission. A total of 108 patients were identified with proton pump inhibitor data from 240 patients in the original cohort. Patients on steroids without proton pump inhibitors were more likely to have a clinical response at week 14 than patients on concomitant PPI (95% vs. 67%, *p* = 0.005). Non-smokers with IBD treated with VDZ were more likely to develop a clinical response at week 14 than smokers, particularly those not receiving PPI compared with patients on co-administered PPI therapy (81% vs. 53%, *p* = 0.041, and 92% vs. 74%, *p* = 0.029, respectively). We found that the use of PPIs in patients treated with VDZ may impair the achievement of response in certain subgroups. Unnecessary PPI prescriptions should be avoided.

## 1. Introduction

Inflammatory bowel disease (IBD) treatment has undergone tremendous progress in recent decades with the introduction of biological therapies. Tumor necrosis factor alpha (TNFα) inhibitors were the first biologic therapies approved in the 1990s. Infliximab (IFX), adalimumab, golimumab, and certolizumab have been shown to be effective in inducing and maintaining both clinical and endoscopic remission of IBD [1,2,3,4]. However, anti-TNF therapy has several limitations, including primary and secondary non-response, immunogenicity, and increased risk of infectious side effects and potential malignancies [1]. This has led to the approval of other, targeted biologic therapies, notably the interleukin-12/23 inhibitor ustekinumab and the gut-selective anti-α4β7-integrin monoclonal antibody vedolizumab (VDZ) [1,5,6,7].

VDZ was approved in the early 2010s for both moderate-to-severe Crohn’s disease (CD) and ulcerative colitis (UC), based on the results of the GEMINI trials (GEMINI 1 for UC, GEMINI 2 and 3 for CD) [5,7,8]. The α4β7 integrin is a cell-surface glycoprotein expressed on circulating T lymphocytes and binds to the mucosal addressin cell adhesion molecule 1 (MAdCAM-1) expressed on intestinal mucosal endothelial cells and gut-associated lymphoid tissue. Inhibition of α4β7 integrins affects the migration of T lymphocytes to the gastrointestinal mucosa during the inflammatory process [9,10]. With this mechanism of action, VDZ exerts its inhibitory effect selectively in the gastrointestinal tract. In contrast, the first anti-integrin natalizumab inhibited both α4β1 and α4β7, thereby also having an inhibitory effect on the central nervous system. The GEMINI LTS trial identified no cases with progressive multifocal leukoencephalopathy (PML) during VDZ exposure [11]. Overall, VDZ has a favorable safety profile, with low incidence rates of serious infections, infusion-related reactions, arthralgia, and nasopharyngitis. However, the risk of infection and serious adverse events was associated with concomitant immunosuppressive therapy (e.g., azathioprine and corticosteroids) [12].

The human gut contains countless organisms, including viruses, bacteria, and fungi, but more than 99% of them belong to four bacterial phyla: *Firmicutes*, *Bacteroidetes*, *Proteobacteria*, and *Actinobacteria* [13,14]. In recent decades, the human microbiome has become one of the main targets of microbiological research, as we know that changes in diversity can be the root cause of many diseases [15]. Recently, a reduced diversity and a disturbed balance between favorable and unfavorable fecal microbiota in IBD have been observed; *Firmicutes* and *Bacteroidetes* in particular are of lower relative abundance [16,17,18,19]. This dysbiosis negatively affects the intestinal immune system and mucosal barrier integrity and may result in the chronic, uncontrollable inflammatory response seen in IBD [20].

Several intrinsic and extrinsic factors can influence the diversity of the microbiome, such as lifestyle, diet, medication, smoking, host genetics, and diseases [21,22]. Recent studies have shown that drugs play a particularly large role in changing the gut microbiome [15]. Above all, antibiotics, proton pump inhibitors, ACE inhibitors, alpha/beta-blockers, and metformin have a significant effect on the composition of the intestinal flora [15]. Proton pump inhibitors (PPIs) are among some of the most commonly prescribed drugs in the world [23]. However, long-term side effects, such as kidney disease, infections, gastrointestinal malignancies, liver disease, fracture risk, cardiovascular disease, myopathy, hypomagnesemia, anemia, and fundic gland polyps, have recently become known [24]. These complications are all the result of a decrease in gastric acid production and thus a change in the diversity of the gut microbiome. Reducing gastric acid production with PPIs can affect the resistance of the gut microbiota to intestinal infections and even promote intestinal inflammation [25,26]. Indeed, the question arises whether the increased use of PPIs may contribute to the increasing incidence of IBD worldwide [27]. Several observational studies and meta-analyses have confirmed that there may be a possible relationship between IBD and PPI intake, so patients exposed to PPI are at significantly increased risk of IBD [28,29]. However, regular PPI users have much higher risk of developing CD than UC [29]. Even the risk of collagenous colitis and lymphocytic colitis seems to be significantly higher with PPI use [28]. In addition, initiation of histamine2-receptor antagonists (H2Ra) doubled and PPIs increased the risk of hospitalization or surgery in patients with CD by 20% [30]. Furthermore, H2Ras stimulate T-cell activity, which can lead to flare ups in CD [30].

Furthermore, many of the current studies evaluating the efficacy of biologics in IBD do not consider the potential confounding effects of concomitant medications. A recently published patient-level meta-analysis of randomized controlled trials showed that patients with IBD taking PPIs were less likely to achieve remission during IFX therapy [31]. The remission rates in patients with and without PPI therapy at weeks 30 and 54 were 30% vs. 49%, and 40% vs. 62%, respectively.

It remains unknown whether PPI use is associated with altered efficacy of VDZ. In order to overcome this lack of knowledge, we aimed to investigate the effect of PPI co-administration in patients with IBD treated with VDZ.

## 2. Materials and Methods

The Hungarian nationwide multicenter VDZ Registry has received ethical approval from the Regional and Institutional Human Medical Biological Research Ethics Committee of the University of Szeged (clinical trial registration number: 99/2017-SZTE) [32]. The study protocol conforms to the ethical guidelines of the Declaration of Helsinki (updated in 2013) [33]. Our current observational, post hoc analysis follows the Strengthening the Reporting of Observational Studies in Epidemiology (STROBE) statement [34] (Table S1).

### 2.1. Design, Setting, and Participants

VDZ (Takeda, Japan) has been available in Hungary since 2016, but the high costs limited treatment options. Therefore, permission from a committee of five Hungarian IBD specialists was required for each patient. The patient applications approved between July 2016 and December 2018 formed the Hungarian nationwide multicenter VDZ cohort. The recommended dose for VDZ induction was 300 mg given as an intravenous infusion at weeks 0, 2, and 6, followed by a maintenance infusion every 8 weeks. Clinical data were prospectively collected from adult patients with moderate and severe disease activity [32]. Concomitant immunosuppressant and corticosteroid treatment was allowed for inclusion, but combination therapy with another biological agent was an exclusion criterion. A total of 240 patients, 127 female and 113 males, received permission for VDZ treatment. In this cohort, 102 patients with CD and 138 with UC were observed and underwent colonoscopies at week 14. Colonoscopies and even data on Mayo or CDAI scores at week 54 were fewer than at week 14 and were incomplete because the patients were unable to continue VDZ at week 54. The primary endpoint of the study was the endoscopic healing rate at the end of short-term (week 14) and long-term (week 54) VDZ treatment [32]. Endoscopic healing rates were significantly higher in UC than in CD patients during short- and long-term therapy (52.9% vs. 21.7%, and 51.4% vs. 21.2%, respectively) [32].

**Figure 1:**
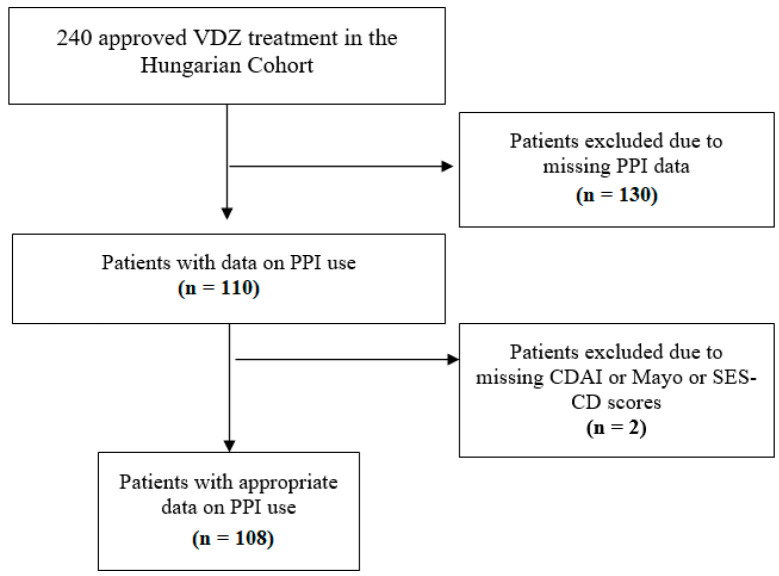
Flow chart of patient selection. VDZ: vedolizumab; PPI: proton pump inhibitor; CDAI: Crohn’s disease activity index; SES-CD: Simple Endoscopic Score for Crohn’s Disease.

In our post hoc analysis, data on PPI use were collected retrospectively. All patients of the Hungarian VDZ cohort who had data about taking PPIs or not were eligible for inclusion. Data on PPI use were obtained from four tertiary care hospitals (Division of Gastroenterology, First Department of Medicine, Medical School, University of Pécs; First Department of Medicine, University of Szeged; Department of Gastroenterology, Hungarian Defence Forces Military Hospital, Budapest; and Balassa János Hospital, Szekszárd). Patients with inadequate data (missing CDAI or Mayo score or endoscopic subscore) were excluded despite PPI treatment (Figure 1).

### 2.2. Outcome Assessment

The primary outcomes of our post hoc analysis were the assessment of clinical response and endoscopic and clinical remission at weeks 14 and 54 during VDZ and PPI co-administration. Clinical response was defined as a reduction of >3 points in the total Mayo score or a reduction of >100 points from baseline in the Crohn’s disease activity index (CDAI). Clinical remission was marked as CDAI score ≤ 150 or Mayo score ≤ 2. Endoscopic remission rates were defined as Simple Endoscopic Score for Crohn’s Disease (SES-CD) score ≤ 4 or Mayo endoscopic subscore ≤ 1. The effect of concurrent steroid therapy and other factors such as smoking on remission was assessed as a secondary outcome.

### 2.3. Bias

**Figure 2:**
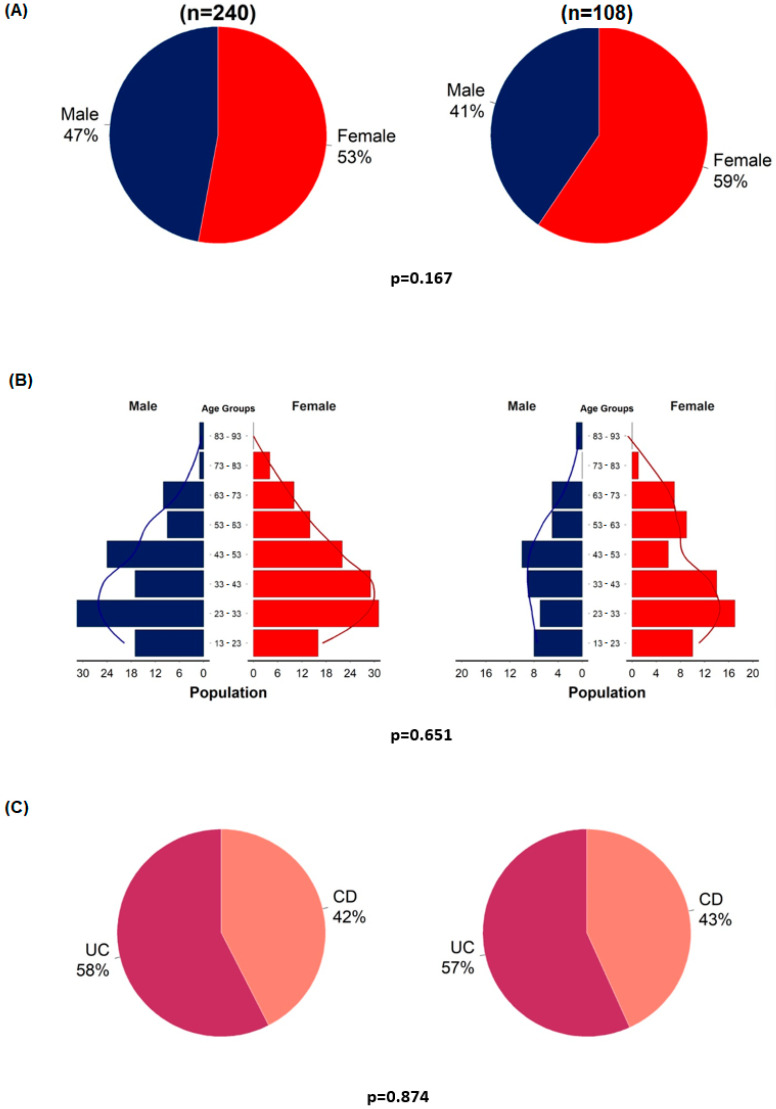
Representativeness analyses of enrolled patients (*n* = 108) compared to the entire cohort (*n* = 240). Gender distribution (**A**); age distribution in males and females (**B**); proportion of patients with ulcerative colitis and Crohn’s disease (**C**).

Before the detailed analyses, representativeness analyses were performed to examine selection bias. Statistical analyses were performed using the entire original cohort and the VDZ–PPI cohort. Gender, age, and the proportion of patients with UC and CD were analyzed (Figure 2).

### 2.4. Statistical Analysis

The data were expressed as mean ± standard deviation (SD) for age and frequencies, and for all other variables as percentages. Independent sample *t*-test and Chi-square test were applied to examine representativeness. Chi-squared tests or Fisher tests were used to explore the relationship between categorical variables, depending on the event rates. Binary logistic regression was performed to identify predictive factors of clinically important outcomes. All calculations were carried out with the R statistical software platform (R version 4.1.2, R Core Team (2021). R: A language and environment for statistical computing. R Foundation for Statistical Computing, Vienna, Austria. [35]).

## 3. Results

The representativeness analyses showed no significant differences between the VDZ–PPI cohort and the entire VDZ cohort in terms of age, gender, and the proportion of CD and UC (Figure 2). Similar to the original cohort, patients with UC were more likely to have a clinical response rate at week 14 than patients with CD (OR = 3.77, 95%CI = 1.3–11.8; *p* = 0.017) (Table S2).

### 3.1. Patient Characteristics

Between 2016 and 2018, 240 patients received approval for VDZ treatment. During patient selection, 130 patients were excluded based on missing PPI data, and another 2 patients were excluded due to lack of endoscopic and CDAI subscores. This left 108 patients in our cohort with data on PPI use for the final analysis (Figure 1).

In the VDZ–PPI cohort, 46 (43%) patients were diagnosed with CD and 62 (57%) with UC (Table 1). The mean age at inclusion was 41 ± 17 years. Only 15% of patients were current smokers (Table 1).

At week 14, 105 and 100 patients had adequate data for clinical remission/response and endoscopic remission, respectively. At week 54, data were available for 75 patients having clinical response, 79 in clinical remission, and 71 in endoscopic remission. 

### 3.2. Main Results

In our cohort, 60 (56%) patients took PPIs and 48 (44%) did not use PPIs. In 47 cases, the PPI prescribed to the patients was pantoprazole (78%), esomeprazole in 7 (12%), omeprazole and lansoprazole in 1-1 (2-2%), and 4 patients (6%) had no data on the type of PPI. When exploring the indications for PPI use, 9 patients took PPIs for GERD, 42 cases for ulcer prevention, and in 9 cases no clear indication was found. Overall, 77% of the patients had concomitant steroid use.

**Figure 3:**
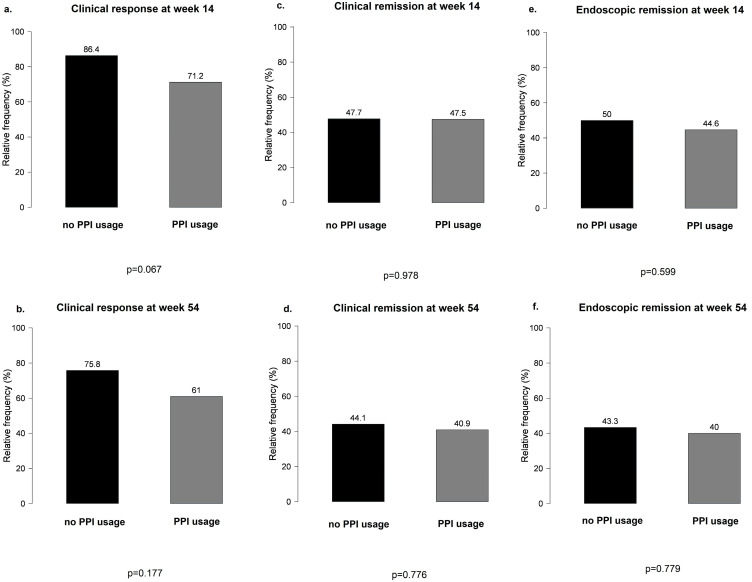
Main results of clinical response and clinical and endoscopic remission between proton pump inhibitor users and non-users during vedolizumab therapy: (**a**,**b**) clinical response at weeks 14 and 54 in PPI users vs. non-users; (**c**,**d**) clinical remission at weeks 14 and 54 in PPI users vs. non-users; (**e**,**f**) endoscopic remission at weeks 14 and 54 in PPI users vs. non-users.

No significant differences were observed between PPI users and non-users during VDZ therapy in terms of clinical response and clinical and endoscopic remission at weeks 14 and 54 (Figure 3).

**Figure 4:**
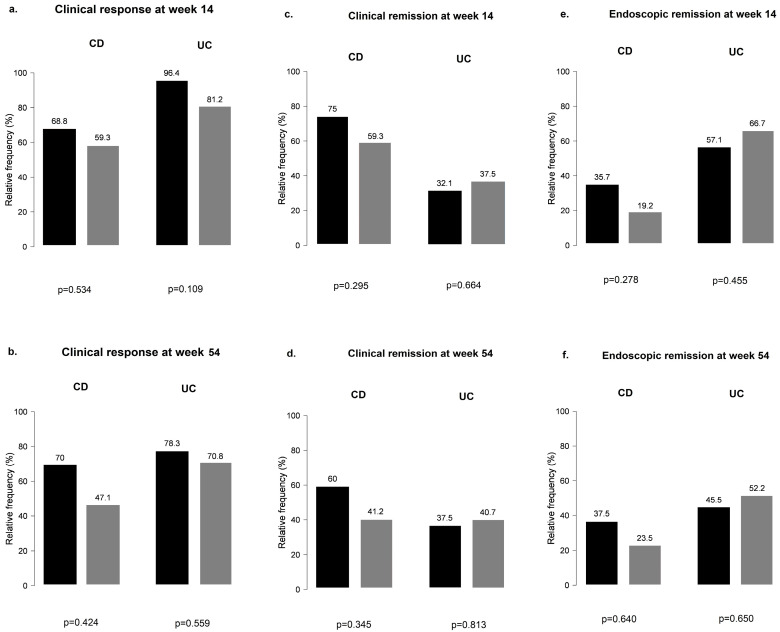
Clinical response and clinical and endoscopic remission outcomes among proton pump inhibitor users and non-users during vedolizumab therapy in Crohn’s disease and ulcerative colitis: (**a**,**b**) clinical response in CD and UC groups with and without the use of PPI; (**c**,**d**) clinical remission in CD and UC groups with and without the use of PPI; (**e**,**f**) endoscopic remission in CD and UC groups with and without the use of PPI; black bar: patients without PPI; grey bar: patients with PPI.

When CD and UC subgroups were analyzed separately for PPI use, results remained non-significant at weeks 14 and 54 for all outcomes (Figure 4).

**Figure 5:**
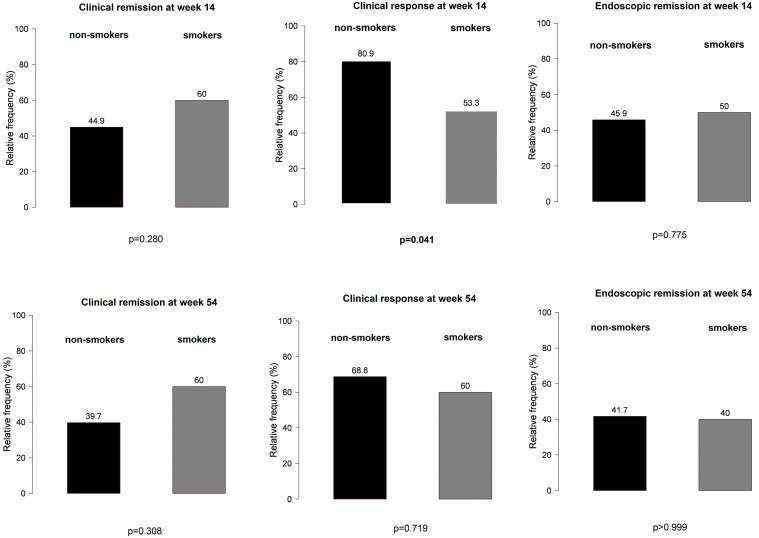
Effect of smoking in clinical response and clinical and endoscopic remission outcomes among patients treated with vedolizumab.

**Figure 6:**
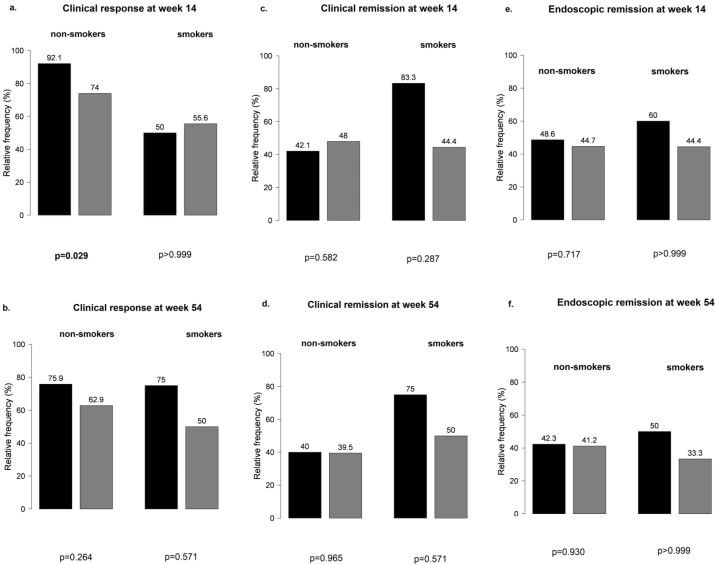
Influence of proton pump inhibitor therapy in smokers and non-smokers treated with vedolizumab: (**a**,**b**) clinical response at weeks 14 and 54; (**c**,**d**) clinical remission at weeks 14 and 5; (**e**,**f**) endoscopic remission at weeks 14 and 54; black bar: patients without PPI; grey bar: patients with PPI.

However, non-smokers with IBD treated with VDZ were more likely to develop a clinical response at week 14 than smoking patients (Figure 5), particularly those not receiving PPI compared with patients on concomitant PPI therapy (81% vs. 53%, *p* = 0.041 and 92% vs. 74%, *p* = 0.029, respectively) (Figure 6). No other significant differences were found between smokers and non-smokers regarding PPI use (Figure 6).

**Figure 7:**
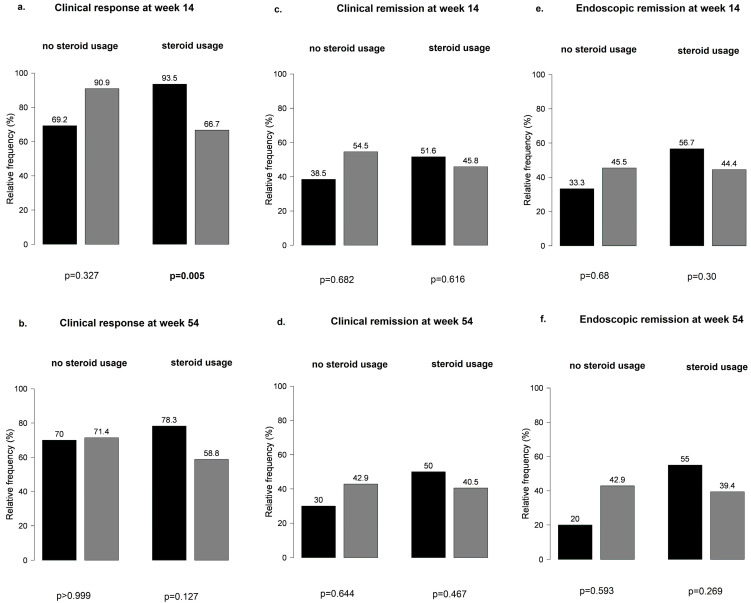
Patients receiving steroid treatment stratified by proton pump inhibitor co-treatment: (**a**,**b**) clinical response at weeks 14 and 54; (**c**,**d**) clinical remission at weeks 14 and 5; (**e**,**f**) endoscopic remission at weeks 14 and 54; black bar: patients without PPI; grey bar: patients with PPI.

Overall, no significant differences were observed at weeks 14 and 54 in the main outcomes between patients with or without steroid treatment (Figure S1). When patients receiving steroid treatment were further stratified by PPI co-treatment, PPI-naïve patients were more likely to have a clinical response at week 14 than patients on PPI therapy (95% vs. 67%, *p* = 0.005) (Figure 7).

## 4. Discussion

During this post hoc analysis of the Hungarian VDZ cohort, we found that the use of PPIs in patients treated with VDZ may impair the achievement of response in certain subgroups. In our cohort, non-smokers treated with VDZ and receiving PPI were less likely to develop a clinical response at week 14 than patients without PPI use. Additionally, less clinical responses were observed at week 14 in patients receiving steroids in whom PPIs were co-administered.

The previous literature has suggested that PPIs increase the risk of IBD in children and adults [29,36]. In a large cohort study, PPI use doubled the risk of hospitalization/surgery in patients with UC or CD [30]. In another study, gastric acid suppression was associated with an increased risk of adverse outcomes in IBD, with PPIs facilitating disease flares [37]. In a recent patient-level meta-analysis of five randomized controlled studies, PPI use was a risk factor for lower remission rates in patients with IBD treated with IFX [31]. There are data to suggest that the microbiota can influence the response to therapies, and that manipulation of the microbiota through fecal transplantation can be used to alter the outcomes of various therapies [38]. Based on previous research, we know that the gut microbiota of patients with IBD is less diverse and contains fewer beneficial bacteria such as *Firmicutes* and *Bacteroidetes* and more pathogenic microorganisms such as *Proteobacteria*, *Escherichia*, and *Fusobacterium* [39,40]. In a recently published systematic review and meta-analysis of double-blind, randomized, controlled trials, clinical and endoscopic benefits were observed for fecal microbiota transplantation in patients with active UC compared to placebo [41].

There is no evidence supporting the routine use of PPIs on patients with IBD as prophylaxis during systemic steroid therapy. In general, steroids may induce well-known gastrointestinal (GI) adverse events, such as peptic ulcer or ulcer bleeding. Therefore, steroids should be prescribed at the lowest effective dose and for the shortest possible duration to minimize side effects.

Another consideration is that PPI treatment is only justified for clearly documented indications. However, in our cohort, the indication for PPI prescription was, in the majority of cases, ulcer prevention. According to NHS advisory guidance, co-prescribing a PPI with steroid therapy is only recommended if high risk factors for steroid-induced GI side effects are present (e.g., older age, history of gastroduodenal ulcer, bleeding or perforation, serious comorbidity, and concomitant medications, such as anticoagulants, antiplatelet agents, and non-steroidal anti-inflammatory drugs) [42]. If patients do not have risk factors, general advice should be provided to help avoid GI adverse effects [42]. Since IBD occurs mainly in young adults, mostly without risk factors for GI adverse events, unnecessary PPI use should be avoided. These recommendations also apply to H2Ras, although they are used less due to the greater efficacy of PPIs.

Accumulating data suggest that long-term use of PPIs can lead, through increased gastric pH and hypochlorhydria, to increased risk of enteric infections such as *Clostridioides difficile* [43]. The potential pathophysiologic link could be that acid suppression alters the gut microbiome and decreases its microbial diversity [44]. Thus, dysbiosis favors the growth of *Clostridioides* species, especially in patients with IBD. Another adverse effect during prolonged use of PPIs can be the occurrence of hypomagnesemia and hypocalcemia, which can be magnified in malabsorption in patients with IBD. The risk of osteoporosis increases with a longer duration of PPI use, especially in post-menopausal women and patients with a history of smoking.

Smoking is considered a risk factor for CD; flare ups and postoperative recurrence are higher in smokers than in non-smokers [45]. In the gut, mucin production, alterations in tight junctions in the small intestine, disruption of gut barrier function, and microbiome changes are induced by cigarette smoking [46,47,48]. There is a clear microbiome signature and reduced gut microbial diversity in patients with CD who smoke versus non-smokers [49]. We hypothesized that there may be a combined effect of smoking and microbial changes due to PPIs during VDZ treatment in IBD. Smoking cessation could contribute to better disease outcomes.

However, we are aware that our cohort study has certain limitations. First, the small number of patients may lead to a bias in the statistics. Second, due to the post hoc nature of data analysis related to PPI use, a clear indication of PPI use (e.g., gastroprotection, gastroesophageal reflux disease) and upper-GI symptoms could not be adequately captured.

The strength of our study is the recruitment of patients from a real-life cohort. This is the first study to report the effect of PPI treatment on the clinical efficacy of gut-selective VDZ treatment.

## 5. Conclusions

In summary, in our post hoc analysis, we found that the concomitant use of PPIs during VDZ treatment may have a negative impact on clinical response in certain subgroups of patients with IBD, i.e., in non-smokers and patients receiving steroid therapy. Therefore, we emphasize avoiding the inappropriate use of PPI in patients with IBD. There is currently no evidence for prophylactic co-prescription of PPIs in order to prevent peptic ulcers in patients without GI risk factors who are taking corticosteroids alone [50]. Long-term use of PPIs taken in the absence of appropriate indications can result in unnecessary side effects, drug interactions, and costs; therefore, the indication for, and duration of, PPI treatment must be considered responsibly in everyday practice.

## Figures and Tables

**Table 1:** Baseline characteristics.

Variable	*n*
Gender	108
Male	44 (41%)
Female	64 (59%)
Age	108
Mean (SD)	41 (17)
Median (IQR)	39 (26, 54)
Smoking	108
no	92 (85%)
yes	16 (15%)
UC/CD	108
CD	46 (43%)
UC	62 (57%)
Steroid	108
no concomitant steroid use	25 (23%)
concomitant steroid use	83 (77%)
PPI	108
no concomitant PPI use	48 (44%)
concomitant PPI use	60 (56%)

## Data Availability

The data presented in this study are available on request from the corresponding author. The data are not publicly available due to the privacy of individuals that participated in the study.

## Supplementary Materials

The following supporting information can be downloaded at https://www.mdpi.com/article/10.3390/biomedicines12010158/s1: Figure S1: Steroid-free remission in patients with vedolizumab treatment; Table S1: Strengthening the Reporting of Observational Studies in Epidemiology (STROBE); Table S2: Clinical response at week 14 (binary logistic regression model).
