# Supplementary material for: Co-Administration of Proton Pump Inhibitors May Negatively Affect the Outcome in Inflammatory Bowel Disease Treated with Vedolizumab
Source: Biomedicines. 2024 Jan 11;12(1):158. doi: 10.3390/biomedicines12010158 (PMC10813460; doi:10.3390/biomedicines12010158)
Supplement: Supplementary file 1 [file biomedicines-12-00158-s001.zip › Suppl. Table S2. Results of logistic regression.pdf]

Supplementary Table S2. Clinical response at week 14: Binary logistic regression model

VARIABLE	N	OR[1]	95% CI[1]	P-VALUE
**AGE**	108	1.02	0.99, 1.05	0.314
**GENDER**	108			
FEMALE	64	1.00	—	
MALE	44	0.911	0.314, 2.69	0.863
**PPI**	108			
NO	48	1.00	—	
YES	60	0.402	0.122, 1.18	0.109
**UC/CD**	108			
CD	46	1.00	—	
UC	62	3.77	1.30, 11.8	**0.017**
**STEROID**	108			
NO	92	1.00	—	
YES	16	0.690	0.174, 2.35	0.570
**SMOKING**	108			
NO	88	1.00	—	
YES	15	0.378	0.104, 1.37	0.132

[1] OR = Odds Ratio, CI = Confidence Interval
