# Supplementary figures and images for: Co-Administration of Proton Pump Inhibitors May Negatively Affect the Outcome in Inflammatory Bowel Disease Treated with Vedolizumab
Source: Biomedicines. 2024 Jan 11;12(1):158. doi: 10.3390/biomedicines12010158 (PMC10813460; doi:10.3390/biomedicines12010158)

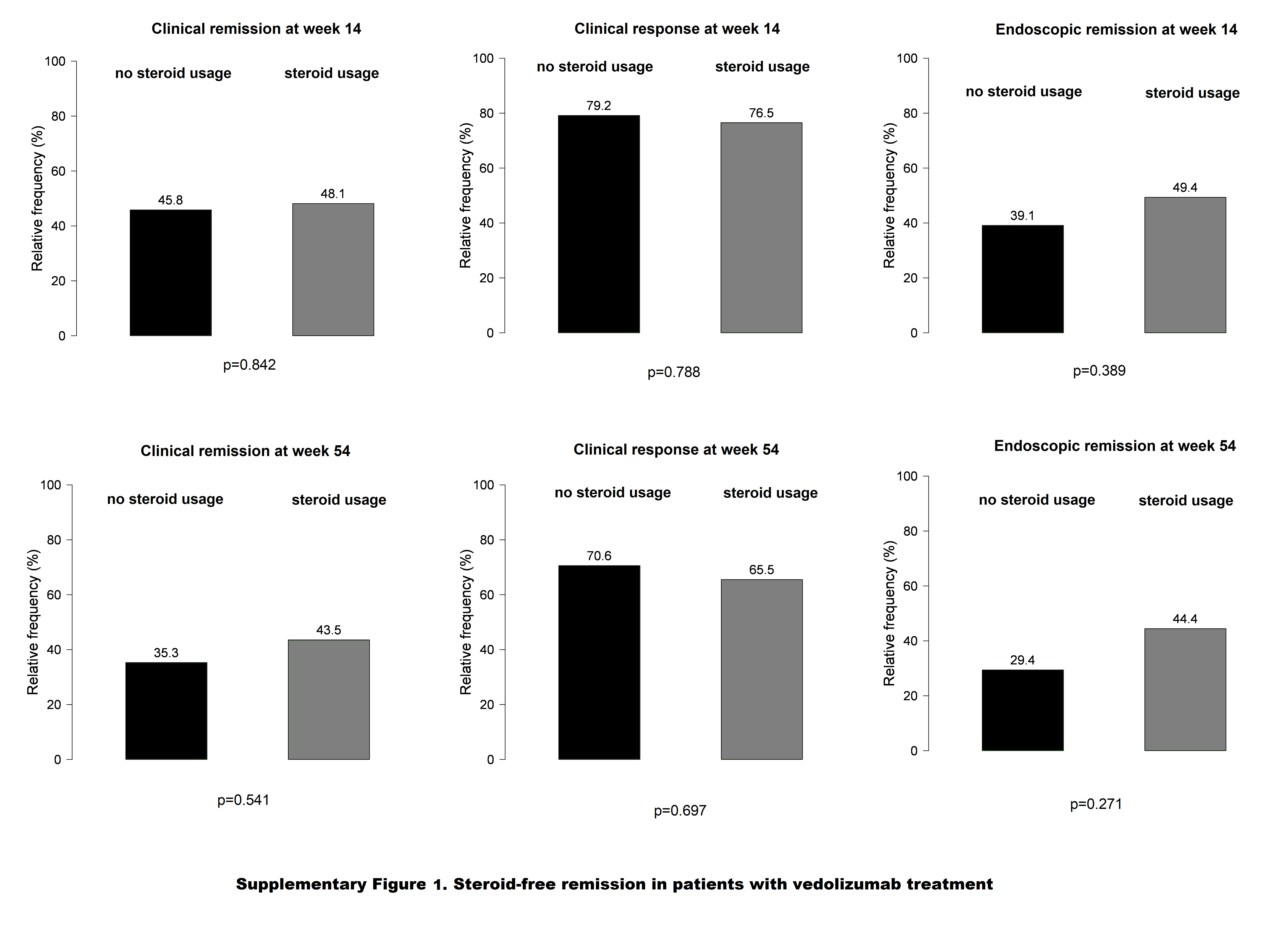

Supplement: Supplementary file 1 [file biomedicines-12-00158-s001.zip › Suppl. Figure S1. Steroid-free remission in patients with VDZ treatment.png]
